# Regulation of subsoiling tillage on the grain filling characteristics of maize varieties from different eras

**DOI:** 10.1038/s41598-021-99916-3

**Published:** 2021-10-14

**Authors:** Li-qing Wang, Xiao-Fang Yu, Ju-Lin Gao, Da-Ling Ma, Liang Li, Shu-Ping Hu

**Affiliations:** Inner Mongolia Agricultural University, No.275, XinJian East Street, Hohhot, 010019 China

**Keywords:** Genetics, Plant sciences

## Abstract

Grain filling is the key stage for achieving high grain yield. Subsoiling tillage, as an effective conservation tillage, has been widely used in the maize planting region of China. This study was conducted to explore the effects of subsoiling on the grain filling characteristics of maize varieties of different eras. Five typical maize varieties from different eras (1970s, 1980s, 1990s, 2000s and 2010s) were used as experimental materials with two tillage modalities (rotation tillage and subsoiling tillage). The characteristic parameters (Tmax: the time when the maximum grouting rate was reached, Wmax: the grain weight at the maximum filling rate, Rmax: the maximum grouting rate, P: the active grouting stage, Gmean: the average grouting rate; A: the ultimate growth mass) and rate parameters (T1: the grain filling duration of the gradually increasing stage, V1: the average grain filling rate of the gradually increasing stage, T2: he grain filling duration of the rapidly increasing stage, V2: the average grain filling rate of the rapidly increasing stage, T3: the grain filling duration of the slowly increasing stage, V3: the average grain filling rate of the slowly increasing stage) of grain filling of two tillage modalities were analyzed and compared. The results showed that the filling parameters closely correlated with the 100-kernel weight were significantly different among varieties from different eras, and the grain filling parameters of the 2010s variety were better than those of the other varieties, the P and Tmax prolonged by 4.06–19.25%, 5.88–27.53% respectively, the Rmax and Gmean improved by 5.68–14.81%, 4.76–12.82% and the Wmax increased by 10.14–32.58%. Moreover, the 2010s variety helped the V2 and V3 increase by 6.49–13.89%, 4.55–15.00%. In compared with rotation tillage, the grain yield of maize varieties from different eras increased by 4.28–7.15% under the subsoiling condition, while the 100-kernel weight increased by 3.53–5.06%. Under the same contrast conditions, subsoiling improved the Rmax, Wmax and Gmean by 1.23–4.86%, 4.01–5.96%, 0.25–2.50% respectively, delayed the Tmax by 4.04–5.80% and extended the P by 1.19–4.03%. These differences were major reasons for the significant increases in 100-kernel dry weight under the subsoiling condition. Moreover, subsoiling enhanced the V2 and V3 by 0.70–4.29%, 0.00–2.44%. The duration of each filling stage and filling rate of maize varieties from different eras showed different responses to subsoiling. Under the subsoiling condition, the average filling rate of the 1970–2010s varieties were improved by 1.18%, 0.34%, 0.57%, 1.57% and 2.69%. In the rapidly increasing period, the grain filling rate parameters of the 2010s variety were more sensitive to subsoiling than those of the other varieties. The rapidly increasing and slowly increasing period are the key period of grain filling. Since the 2010s variety and subsoiling all improve the grain filling rate parameters of two periods, we suggest that should select the variety with higher grain filling rate in the rapidly increasing and slowly increasing period, and combine subsoiling measures to improve the grain filling characteristic parameters of maize in production, so as to achieve the purpose of increasing 100 grain weight and yield.

## Introduction

Previous study about maize high yield home and abroad show that increasing maize yield requires sufficient water and fertilizer, high yield and density tolerant varieties, high planting density and reasonable cultivation measures^1,2^. Therefore, soil fertility improvement, maize variety improvement and innovative cultivation techniques have become effective ways to increase the maize yield per unit area in the context of the rigid demand for maize grain yield, the reduction of cultivated land and water shortage in China.

The grain filling stage is the key period for grain matter accumulation and yield formation of maize. Increasing planting density will decline the photosynthetic characteristics, which resulted in grain filling rate and grain weight decreasing, inevitably leading to a decline in grain yield per plant, and the disadvantages of topsoil structure further affect the plant population capacity^3–5^. A tillage layer with good structure helps plants growth, alleviates the adverse effects of increased density, and practices that optimize topsoil structure are key measures for improving maize yield in China. As a conservation tillage measure, subsoiling can effectively improve soil physical and chemical properties, enhance plant self-regulation ability, promote maize root penetration, increase the photosynthetic rate, and delay leaf senescence, as to alleviate the cluster effect of the planting density, effectively stabilize population yield and realize planting density and yield improvement^6–9^.

Previous works showed that grain filling was an important physiological process that determined the yield and quality of maize grain^10,11^. Grain filling can be optimized by appropriate tillage practices^12,13^, varieties^10,14^, planting density^15,16^ and management measures^17^, as well as constructing a reasonable canopy structure to realize full utilization of light, heat, water and fertilizer. Good filling conditions can achieve coordination of ear number, ear grain number and grain weight, increasing the maize yield per unit area.

Studies have shown that subsoiling can improve the maize yield by increasing planting density. However, few studies have assessed the effects of subsoiling on maize grain filling characteristics. Therefore, maize varieties from different eras were used as experimental materials to study the effects of subsoiling on grain filling characteristics. The results will provide a theoretical basis for further exploration of the mechanisms of increasing yield by subsoiling.

## Materials and methods

### Trials and measurements

**Figure 1:**
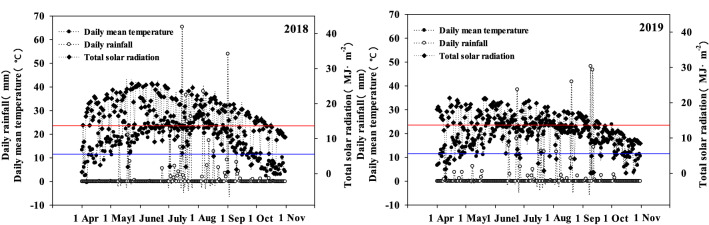
Main meteorological factors during the growth period in the experimental area.

Field experiments were carried out at the Tumoteyou Qi Experimental Station of the Inner Mongolia Agricultural University (40°33′N, 110°31′E) during 2018 and 2019, where its loam has a 0–30 cm soil layer containing 22.27 g kg^−1^ organic matter, 103.75 mg kg^−1^ available nitrogen, 15.76 mg kg^−1^ available phosphorus, and 219.60 mg kg^−1^ available potassium (pH 8.23). The main meteorological factors during the maize growth period are given in Fig. 1.

The experiment adopted a two-factor split-plot design (tillage treatment and variety). Tillage treatment was the main plot, including subsoiling (SS) with a depth of 35 cm and rotary tillage (RT) with a depth of 15 cm; the subplots were 1970s–2010s maize varieties: ZD2 (1970s), DY13 (1980s), YD13 (1990s), XY 335 (2000s), and DH618 (2010s). These varieties are sold in Chinese markets and were purchased as test materials. Each subplots repeated three times, the planting density was 75,000 plants ha^−1^ with row spacing of 0.6 m. The plot area was 6 m × 6 m. The dosages of N, P_2_O_5_ and K_2_O were 465 kg ha^−1^, 210 kg ha^−1^, and 202.5 kg ha^−1^. P_2_O_5_ and K_2_O were applied as basal fertilizer at seeding. At V6 (sixth leaf), V12 (twelfth leaf), and R2 (blister), N was applied as fertilizer at the ratio of 3:6:1. Subsoiling was achieved with a five-shovel subsoiling plough, and a John Deere 1654 tractor. The plots were irrigated four times during the growth period (seeding stage, V12, R1 (silking) and R2) at 750 m^3^ ha^−1^. The main soil physical characteristic indexes in the trial area are given in Table 1.

**Table 1:** Main soil physical properties in the test area.

Years	Tillage method	Emergence (VE)		Milk stage (R3)	
		Soil compaction (Kpa)	Soil moisture content (%)	Soil compaction (Kpa)	Soil moisture content (%)
2018	Rotary tillage (RT)	2194.19	16.59	2675.5	14.94
	Subsoiling (SS)	1775.23	19.23	2443.14	16.11
2019	Rotary tillage (RT)	2352.46	16.97	1333.41	18.84
	Subsoiling (SS)	1850.77	14.01	1143.86	15.90

### Measurement

#### Grain filling characteristics

From 15 days since pollination, samples were collected at 3-day intervals until the end of filling. At each sampling point, three ears were collected per plot, and 100 kernels were collected from the middle of each ear. The kernels were weighed, placed into an oven for 30 min at 105 °C, dried at 80 °C to a constant weight, and weighed again^10^. The procedures for the collection of experimental material complied with relevant institutional, national, and international guidelines and legislation.

A logistic equation^18^ was used to fit the grain filling process, calculate grain filling characteristic parameters, and analyze grain filling growth. The logistic equation was as follows:

$${\text{W}} = {{\text{A}} \mathord{\left/ {\vphantom {{\text{A}} {\left( {1 + {\text{Be}}^{{ - {\text{Ct}}}} } \right)}}} \right. \kern-\nulldelimiterspace} {\left( {1 + {\text{Be}}^{{ - {\text{Ct}}}} } \right)}}$$

In the equation above, *t* is the number of days after flowering (blooming day t_0_ = 0), *w* is the 100-kernel weight after flowering (grain weight on flowering day = w_0_), *A* is the theoretical maximum 100-kernel weight, and *B* and *C* are shape parameters. The filling parameters were derived from the first and second derivatives of the equation.

- t_1_ (the start date of the filling peak period) = (lnB − 1.317)/C, corresponding to the grain weight (w_1_) at this time: w_1_ = A/(1 + Be^−Ct1^);
- t_2_ (the end date of the filling peak period) = (lnB + 1.317)/C, corresponding to the grain weight (w_2_) at this time: w_2_ = A/(1 + Be^−Ct2^);
- t_3_ (the grain weight reaches 99% after flowering, the effective filling period = (lnB + 4.59512)/C, corresponding to the grain weight (w_3_) at this time.

The filling duration of the gradually increasing period was calculated as T_1_ = t_1_ − t_0_. The increase in grain weight during the rapidly increasing period was calculated as w_1_ = W_1_ − W_0_. The mean filling rate of the gradually increasing period was calculated as V_1_ = w_1_/T_1_.

The filling duration of the rapidly increasing period was calculated as T_2_ = t_2_ − t_1_. The increase in grain weight during the rapidly increasing period was calculated as w_2_ = W_2_ − W_1_. The mean filling rate of the rapidly increasing stage was calculated as V_2_ = w_2_/T_2_.

The filling duration of the slowly increasing period was calculated as T_3_ = t_3_ − t_2_. The increase in grain weight of the slowly increasing period was calculated as w_3_ = W_3_ − W_2_. The mean filling rate of the slowly increasing stage was calculated as V_3_ = w_3_/T_3_;

The final grain growth was A. Tmax (maximum filling rate time) = lnB/C, Wmax (the grain weight at the maximum filling rate) = A/2, Rmax (the maximum filling rate) = (CWmax)·(1 − Wmax/A), P (time to complete approximately 90% of total accumulation) = 6/C, and Gmean (the mean filling rate) = W_3_/t_3._

#### Determination of grain weight

At physiological maturity, ten ears were randomly selected from each plot and air-dried. 100 kernels were then collected from the middle of each ear and weighed, and this weight was converted into the 100-grain weight with 14% moisture content^19^. The determination of grain weight complied with the GB/T 5519-2008 national standard.

### Statistical analysis

Statistical analysis was performed using Microsoft Excel 2016 (Microsoft, Inc., red-mond WA, USA, https://www.microsoft.com/zh-cn/download/office.aspx) and SAS 9.4 statistical software (SAS Institute Inc., CA, USA, https://brand.sas.com/en/home.html). The factor analysis was carried out using IBM SPSS Statistics 25.0 (IBM inc., Armonk, NY, USA, https://www.ibm.com/cn-zh/analytics/spss-statistics-software). The filling dynamic fitting was carried out using Graph Pad Prism 5.0 software (Graph Pad Software inc., San Diego, CA, USA, https://www.graphpad.com/), and SigmaPlot 12.5 (Systat Software Inc., San Jose, CA, USA, https://systatsoftware.com/) was used to create figures.

## Results

### Effect of subsoiling on the 100-kernel weight and grain yield

Analysis of variance showed that the effects of different tillage methods, varieties and years on the 100-grain weight were significant at *p* < 0.01, but the effect of tillage method*variety was not significant. The tillage methods and varieties had significant effects on the grain yield (*p* < 0.01), but the effect of years and tillage method*variety were not significant (Table 2).

**Table 2:** Variance analysis of the effect of tillage method and variety on the 100-grain weight and grain yield of maize.

Source	DF	Mean square	
		100-grain weight	Grain yield
Tillage method (M)	1	38.53**	7.45**
Main area error	2	1.27	0.07
Variety (V)	4	181.46**	48.92**
V*M	4	0.40	0.01
Secondary area error	16	0.54	0.19
Years	1	7.95**	1.99

**Significant at *P*, 0.01.

**Figure 2:**
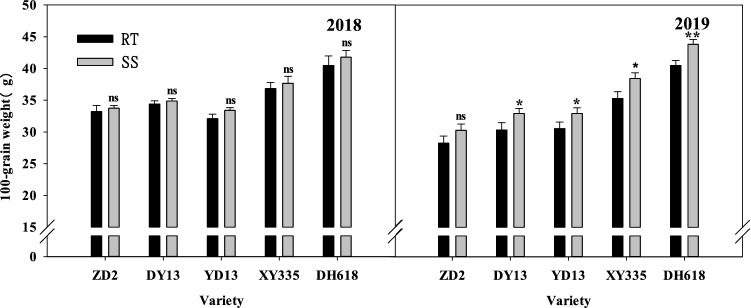
Effects of subsoiling tillage on the 100-grain weight of different ages maize varieties. *,** and ns represent the ANOVA analysis between RT and SS, *Significant at *P*, 0.05. **Significant at *P*, 0.01. “ns” no significant at *P*, 0.05.

Under the rotation tillage (RT) condition, the mean grain weight of each of the 1970s–1990s varieties was lower than that of the 2010s variety, and the 100-kernel weight of DH618 (2010s) significantly increased (*P* < 0.05). Compared with ZD2, the 100-kernel weight of DY13, YD13, XY335, and DH618 increased by 1.17, − 1.14, 3.62 and 7.24 g respectively in 2018, and increased by 1.81, 1.99, 6.07 and 10.50 g in 2019 (Fig. 2).

Subsoiling tillage increased the 100-kernel weight of each variety. However, there were differences in the responses of the varieties to subsoiling. Compared with RT, the 100-kernel weight of ZD2, DY3, YD13, XY335 and DH618 increased by 0.52, 0.47, 1.31, 0.82 and 1.31 g respectively in 2018, and increased by 1.76, 2.23, 2.06, 2.68 and 2.86 g in 2019, (*P* < 0.05), other varieties reached a significant level except ZD2.

**Figure 3:**
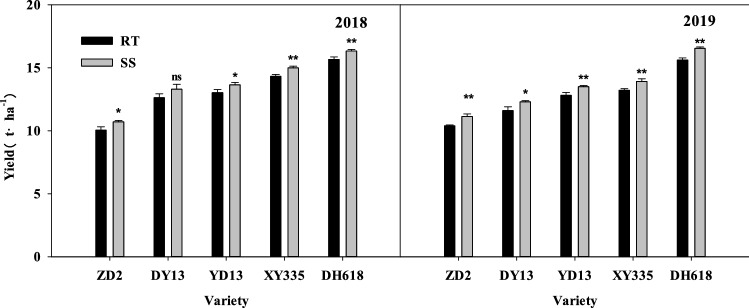
Effects of subsoiling tillage on the yield of different ages maize varieties. *,** and ns represent the ANOVA analysis between RT and SS, *Significant at *P*, 0.05. **Significant at *P*, 0.01. ns no significant at *P*, 0.05.

The variation of the yield was basically consistent with the 100-grain weight. Under the rotation tillage (RT) condition, the increasing of maize yield as the developing of originating varieties became more obvious. The yield of DH618 (2010s) has increased significantly (*P* < 0.05) by 5.62, 3.03, 2.64 and 1.34 t ha^−1^ respectively compared with the 1970s–2000s varieties in 2018, and increased by 5.22, 4.01, 2.80 and 2.40 t ha^−1^ than those in 2019 (Fig. 3).

Subsoiling tillage contributed to the increase of the yield of each variety, but obvious differences existed among the varieties. Compared with RT, the yield of ZD2, DY13, YD13, XY335 and DH618 have increased by 0.66, 0.68, 0.63, 0.67 and 0.67 t ha^−1^ respectively in 2018 and by 0.74, 0.70, 0.68, 0.70 and 0.92 t ha^−1^ in 2019.

### Effect of subsoiling on the kernel dry matter accumulation

**Figure 4:**
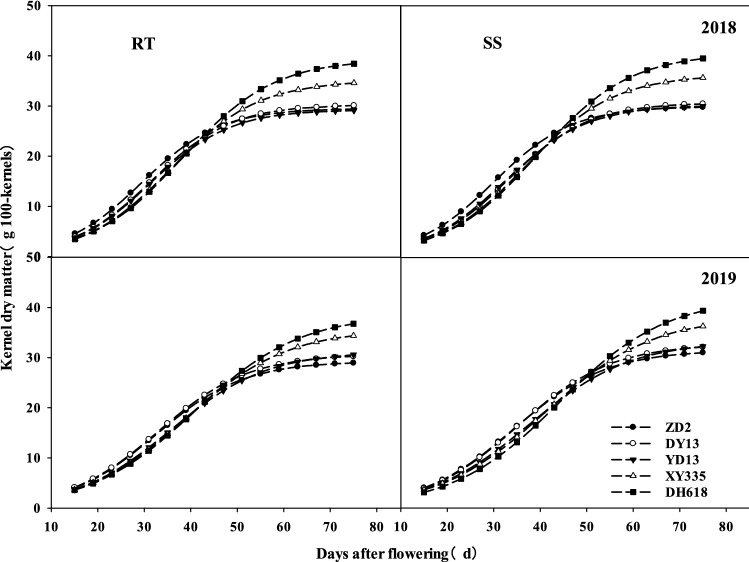
Effects of subsoiling tillage on the dry weight of 100 grains of maize varieties from different era.

As shown in Fig. 4, the kernel dry weight started to gradually increase since 15th day after silking, and achieved maximum dry weight at physiological maturity. The kernel dry weight of the tested varieties showed no significant difference within 0–43 days after flowering; however, as the filling process continued, the difference emerged progressively. The kernel weight of ZD2, DY13, YD13, XY335, and DH618 increased by 24.66, 23.91, 23.34, 24.07 and 24.49 g, respectively, within 0–43 days after flowering, and increased by 4.74, 6.19, 5.76, 10.52 and 13.95 g, within 43–75 days after flowering in 2018. In 2019, their kernel weight increased by 22.03, 22.54, 20.91, 21.18 and 21.16 g, respectively, within 0–43 days after flowering, and increased by 6.89, 7.69, 9.67, 13.19 and 15.60 g, within 43–75 days after flowering. These results showed that the period of 43–75 days after flowering was the main stage during which differences between the kernel dry weight of the old and new varieties were observed.

Compared with RT, subsoiling tillage improved the kernel dry weight of the tested varieties, but the extent of the improvement was inconsistent for different filling processes. The kernel weight of ZD2, DY13, YD13, XY335, and DH618 changed showed little significance by − 0.03, − 0.64, − 0.10, − 0.33 and − 0.58 g respectively from 0 to 43 days after flowering in 2018. However, within 43–75 days after flowering, increased by 0.43, 1.00, 1.01, 1.38 and 1.65 g. In 2019, their kernel weight showed little significance by 0.18, − 0.10, − 0.23, − 0.53 and − 1.13 g within 0–43 days after flowering, increased by 1.84, 1.94, 1.85, 2.38 and 3.69 g within 43–75 days after flowering. These results showed that subsoiling mainly boosted the increase of the kernel dry weight within 43–75 days after maize flowering, and the effect became more obvious as the developing of originating varieties.

### Effect of subsoiling on the filling characteristic of maize varieties from different eras

#### The filling characteristic parameters

As shown in Table 3, the trends of the filling characteristic parameters data in the 2-year trial were basically the same under the RT and SS conditions. The filling parameters tended to increase, decrease, and then increase again during variety replacement with YD13 showing the lowest values, and DH618 showing the highest values. Therefore, the data in 2018 and 2019 were averaged for the following analysis. Under the RT condition, the *A* values of ZD2, DY13, YD13, XY335 and DH618 were 29.35, 30.42, 30.19, 35.32 and 38.91 g, respectively; the Tmax values were 30.91, 32.25, 33.44, 37.23 and 39.42 days; the Wmax values were 14.67, 15.21, 15.10, 17.66 and 19.45 g 100-kernel^−1^; the Rmax values were 0.83, 0.84, 0.81, 0.88 and 0.93 g 100-kernel^−1^ days^−1^; the *P* values were 53.09, 54.45, 56.02, 60.84 and 63.31 days; the Gmean values were 0.41, 0.41, 0.39, 0.42 and 0.44 g 100-kernel^−1^ days^−1^. It could be perceived that all of the filling parameters of the modern varieties were improved.

**Table 3:** Response of the grain filling characteristic parameters of maize varieties from different eras to tillage methods.

Year	Tillage method	Variety	A (g)	Tmax (days)	Wmax (g 100-kernel^−1^)	Rmax (g 100-kernel^−1^ days^−1^)	P (days)	Gmean (g 100-kernel^−1^ days^−1^)
2018	RT	ZD2	29.48	29.36	14.74	0.88	49.99	0.432
		DY13	30.26	31.47	15.13	0.88	51.39	0.423
		YD13	29.24	31.22	14.62	0.86	50.86	0.413
		XY335	35.04	35.64	17.52	0.94	56.16	0.441
		DH618	39.33	38.07	19.66	1.00	58.99	0.468
	SS	ZD2	29.89	30.08	14.95	0.90	49.86	0.433
		DY13	30.68	33.07	15.34	0.89	51.61	0.418
		YD13	30.20	32.47	15.10	0.87	52.14	0.413
		XY335	36.23	36.96	18.12	0.96	56.52	0.447
		DH618	40.56	39.40	20.28	1.02	59.46	0.473
2019	RT	ZD2	29.21	32.46	14.60	0.78	56.18	0.383
		DY13	30.57	33.02	15.29	0.80	57.51	0.393
		YD13	31.13	35.66	15.57	0.76	61.17	0.374
		XY335	35.60	38.82	17.80	0.81	65.52	0.396
		DH618	38.48	40.77	19.24	0.85	67.62	0.412
	SS	ZD2	31.40	34.24	15.70	0.79	59.27	0.390
		DY13	32.60	35.11	16.30	0.82	59.60	0.400
		YD13	33.20	37.51	16.55	0.77	64.40	0.377
		XY335	38.02	40.98	19.01	0.83	68.41	0.403
		DH618	41.89	44.01	20.94	0.92	68.66	0.429

Tmax is the time when the maximum grouting rate was reached; Wmax is the grain growth at the maximum filling rate; Rmax is the maximum grouting rate; P is the active grouting stage; Gmean is the average grouting rate; A is the ultimate growth rate of the grain.

Compared with rotary tillage, subsoiling witnessed a significantly increase of the grain filling characteristic parameters of maize varieties from different eras. The *A* values of ZD2, DY13, YD13, XY335 and DH618 increased by 4.43%, 4.03%, 5.02%, 5.11%, 5.96%, respectively; the Wmax values increased by 4.46%, 4.01%, 4.84%, 5.12%, and 5.96%; the Rmax values increased by 1.81%, 1.79%, 1.23%, 2.29%, and 4.86%; the *P* values increased by 2.79%, 2.12%, 4.03%, 2.67%, and 1.19%; the Gmean values increased by 1.13%, 0.29%, 0.55%, 1.56%, and 2.59%. These results indicated that subsoiling can effectively regulate the filling characteristic parameters of maize varieties from different eras, and the effect of subsoiling became more obvious as the developing of originating varieties.

#### Path analysis of grain filling characteristic parameters and 100-kernel weight

In order to clarify the direct and indirect relationships between grain filling parameters and maize grain kernel weight, path-coefficient analysis was performed (Table 4). The result showed that the Tmax, Wmax and *P* were significantly positively correlated with 100-kernel weight (*P* < 0.01), and the Rmax and Gmean were positively correlated with 100-kernel weight (*P* < 0.05). The correlation coefficients were ranked as follows: Wmax > Tmax > P > Rmax > Gmean. There was directly positive correlation between Wmax and 100-kernel weight with the correlation coefficient being 0.939. It showed indirectly positive correlations between Tmax, Rmax, *P* and Gmean and 100-kernel weight through Wmax, and their correlation coefficients were 0.866, 0.495, 0.667, and 0.484. Comprehensive analysis indicated that cultivation tillage can postpone the appearance of maximum filling rate, and increase the maximum filling rate, and thus increase kernel growth, ultimately increasing 100-kernel weight.

**Table 4:** Path analysis of grain filling characteristic parameters.

Index	Correlation coefficient	Direct path coefficient	Coupling diameter factor				
			X1	X2	X3	X4	X5
X1	0.893**	0.209		0.866	− 0.021	− 0.174	0.013
X2	0.976**	0.939	0.193		− 0.059	− 0.138	0.041
X3	0.540*	− 0.113	0.039	0.495		0.040	0.079
X4	0.683**	− 0.191	0.191	0.677	0.023		− 0.017
X5	0.527*	0.080	0.033	0.484	− 0.111	0.041	

X1 stands for Tmax, X2 stands for Wmax, X3 stands for Rmax, X4 stands for P, X5 stands for Gmean, and Y stands for 100-grain weight. Tmax is the time when the maximum grouting rate was reached; Wmax is the grain growth at the maximum filling rate; Rmax is the maximum grouting rate; *P* is the active grouting stage; Gmean is the average grouting rate; A is the ultimate growth rate of the grain.

#### The filling rate parameters

The durations of each stage of maize grain filling were ranked as follows: slowly increasing stage > rapidly increasing stage > gradually increasing stage. The mean filling rates of each stage were ranked as follows: rapidly increasing stage > gradually increasing stage > slowly increasing stage (Table 5). Except for the grain filling rate of the gradually increasing stage, other grain filling rate parameters increased with maize variety replacement. Under RT, the 2-year mean T1 of ZD2, DY13, YD13, XY335, and DH618 were 19.26, 20.30, 21.14, 23.87 and 25.52 days, respectively; T2 were 23.31, 23.91, 24.59, 26.71 and 27.79 days; T3 were 29.01, 29.75, 30.61, 33.24 and 34.59 days. Their V1 values were 0.33, 0.32, 0.31, 0.32 and 0.32 g 100-kernel^−1^ days^−1^, V2 values were 0.73, 0.74, 0.72, 0.77 and 0.82 g 100-kernel^−1^ days^−1^, and V3 values were 0.21, 0.21, 0.20, 0.22 and 0.23 g 100-kernel^−1^ days^−1^.

**Table 5:** Response of the grain filling rate parameters of maize varieties from different eras to tillage methods.

Year	Tillage method	Variety	T1 (days)	V1 (g 100-kernel^−1^ days^−1^)	T2 (days)	V2 (g 100-kernel^−1^ days^−1^)	T3 (days)	V3 (g 100-kernel^−1^ days^−1^)
2018	RT	ZD2	18.39	0.34	21.95	0.78	27.31	0.22
		DY13	20.19	0.32	22.56	0.77	28.08	0.22
		YD13	20.05	0.31	22.33	0.76	27.79	0.21
		XY335	23.31	0.32	24.65	0.82	30.68	0.23
		DH618	25.12	0.33	25.90	0.88	32.23	0.25
	SS	ZD2	19.14	0.33	21.89	0.79	27.24	0.22
		DY13	21.74	0.30	22.66	0.78	28.20	0.22
		YD13	21.03	0.30	22.89	0.76	28.48	0.21
		XY335	24.55	0.31	24.81	0.84	30.88	0.24
		DH618	26.35	0.33	26.10	0.90	32.49	0.25
2019	RT	ZD2	20.13	0.31	24.66	0.68	30.70	0.19
		DY13	20.40	0.32	25.25	0.70	31.42	0.20
		YD13	22.23	0.30	26.85	0.67	33.42	0.19
		XY335	24.43	0.31	28.77	0.71	35.80	0.20
		DH618	25.92	0.31	29.68	0.75	36.94	0.21
	SS	ZD2	21.23	0.31	26.02	0.70	32.38	0.20
		DY13	22.03	0.31	26.17	0.72	32.57	0.20
		YD13	23.37	0.30	28.27	0.68	35.18	0.19
		XY335	25.96	0.31	30.03	0.73	37.37	0.20
		DH618	28.94	0.31	30.14	0.80	37.51	0.22

T1 represents the grain filling duration of the gradually increasing stage, V1 represents the average grain filling rate of the gradually increasing stage, T2 represents the grain filling duration of the rapidly increasing stage, V2 represents the average grain filling rate of the rapidly increasing stage, T3 represents the grain filling duration of the slowly increasing stage, and V3 represents the average grain filling rate of the slowly increasing stage.

Under the subsoiling condition, the grain filling durations of different grain filling stages were all prolonged in comparison with RT, with the gradually increasing stage being the longest. The averaged two-year data revealed that the filling durations of ZD2, DY13, YD13, XY335, and DH618 were prolonged by 4.80%, 7.83%, 5.01%, 5.80% and 8.33% respectively in the gradually increasing stage. In addition, subsoiling helped increase the filling rate of ZD2, DY13, YD13, XY335, and DH618 by 2.05%, 2.04%, 0.70%, 2.61%, and 4.29% in the rapidly increasing stage, but it had little effect on the filling rate during other stages. These results indicated that subsoiling mainly extended the filling duration of the gradually increasing stage and improved the filling rate during the rapidly increasing stage. Moreover, maize varieties from more recent eras were found to be more sensitive to the effect of subsoiling in comparison with relatively older varieties.

#### Analysis of the grain filling rate parameter factor

In order to clarify the internal dependence of the grain filling rate and duration at each stage, factor analysis was performed using the two-year trial data (Table 6). Under RT condition, the factor load difference of the filling duration was small at each stage (T1 = 0.96, T2 = 0.96, T3 = 0.96), which indicated that the proportion of the filling duration of each stage was nearly identical. Under SS condition, it showed a large factor load difference of the grain filling duration between T1 and T2/T3 (T1 = 0.91, T2 = 0.97, T3 = 0.97), which indicated that the effect of subsoiling on the grain filling duration was stronger during the rapidly increasing and slowly increasing stages. In addition, the factor load of the filling rate during the rapidly and slowly increasing stages was higher than that of the gradually increasing stage, which showed that the filling rate during the rapidly and slowly increasing stages contributed more to the mean filling rate in comparison with that of the gradually increasing stage. These results demonstrate that appropriate tillage measures can improve the filling rate during the rapidly increasing and slowly increasing stages, and thus improve the mean filling rate.

**Table 6:** Parameter factor analysis of grain filling rate.

Tillage methods	RT		SS	
Index	Y1	Y2	Y1	Y2
T1	0.961	0.249	0.914	0.340
V1	− 0.414	0.766	− 0.055	0.721
T2	0.958	− 0.233	0.970	− 0.211
V2	0.021	0.985	− 0.001	0.983
T3	0.958	− 0.234	0.971	− 0.210
V3	0.022	0.992	− 0.069	0.971

The extraction method was principal component analysis, Y1 represents the grouting duration, and Y2 represents the average grouting rate. T1 represents the grain filling duration of the gradually increasing stage, V1 represents the average grain filling rate of the gradually increasing stage, T2 represents the grain filling duration of the rapidly increasing stage, V2 represents the average grain filling rate of the rapidly increasing stage, T3 represents the grain filling duration of the slowly increasing stage, and V3 represents the average grain filling rate of the slowly increasing stage.

## Discussion

Previous studies have shown that the grain weight of maize was mainly determined by filling rate and filling duration. Therefore, it could increase grain weight and yield by improving grain filling rate and ensuring that grain filling was maintained for an appropriate duration^20–23^. Fang et al.^24^ suggested that the grain filling rate determined dry matter accumulation and yield, and that the implementation of a reasonable planting method could improve grain filling rate. Gasura et al.^25^ reported that extending the active grain filling period and increasing the mean filling rate could increase maize yield. Daynard et al.^26^ found that prolonging the filling duration help increase the 100-kernel weight. On the basis of previous studies, this study found that improving mean filling rate and prolonged filling duration of each grain filling stage could contributed to grain growth. In addition, we found that the Wmax was boosted by improving Rmax and prolonged Tmax and therefore increase the 100-kernel weight. In addition, this study explored that mean filling rate was determined primarily by filling rates of the rapidly increasing and slowly increasing stages. Therefore, the level of plant material accumulation during the period from 20 days after silking to physiological maturity (including the rapidly increasing and slowly increasing stages of grain filling) was strongly correlated with the mean filling rate. Their interaction help maximize plant dry matter accumulation, which will be beneficial to the further improvement of grain weight.

In view of the close relationship between 100 grain weight and grain filling characteristics, extensive researches have also been done by scholars around the world. The results showed that the grain filling rate of high-yield varieties was significantly higher than the common varieties^27^, since the duration of grain filling was easily limited by local ecological conditions and planting density. On the basis of ensuring a certain filling duration, increasing the grain filling rate and accelerating the accumulation of assimilation in the grain will have a more significant effect on improving yield. Wang^18^ suggested that under the premise of ensuring the safe maturity of grains, extending the duration of grain filling at active stage and improving the filling rate at increasing stage will be conducive to improving the yield of maize at different maturity stages. Li^28^ also found that contemporary parental inbred lines had high dry matter accumulation and daily increased dry weight. This study showed that various grains filling characteristic parameters of modern varieties (DH618) increased, especially Tmax and Gmean change more. During the experiment, we observed that the reason for prolonging Tmax of modern varieties (DH618) was that the silking period was significantly earlier. We also found that the increase in Gmean of the modern varieties (DH618) was mainly due to the increase in V2 and V3. But this part of the study was different from the results of Wang et al., which may be due to the difference in maturity of the tested varieties.

Conservation agriculture, such as the practice of no tillage, less tillage, and straw mulch, has been an important strategy for the sustainable development of worldwide agriculture in the past few decades because it can improve soil properties while increasing crop yields and farmer income^29^. Farming methods are a key factor affecting soil systems in China, where soil management and seeding are mainly performed with small tractors involving less tillage or no tillage, which increase the surface soil bulk density and osmotic resistance, which have deleterious effects on crop growth^30–32^. Previous studies demonstrated that optimized farming boosted crop yield^33,34^. For example, subsoiling increased yield by improving 100-kernel weight of maize^35^. Zhai et al.^36^ showed that subsoiling increased Rmax and Gmean so that the maximum kernel weight was increased significantly at harvest. Cai et al.^37^ showed that subsoiling increased kernel weight, improved plant resistance to environmental stresses, and increased yield. In this study, we further analyzed the filling characteristics of maize varieties from different eras in subsoiling. The results showed that subsoiling increased the kernel weight by improving Tmax, Wmax, P and A, as well as significantly improved the Rmax and Gmean compared with conventional rotation. The beneficial effects of subsoiling on filling characteristics were likely observed because subsoiling tillage breaked the ploughed stratum, increased the topsoil depth, improved water storage and moisture conservation, promoted root growth and development^38,39^, maintained a high leaf area index and photosynthetic rate after anthesis, and helped to produce more photosynthetic products^40^, thus increasing the grain filling rate and kernel weight.

## Conclusion

The grain filling is a key factor in grain yield formation, and the kernel weight and grain yield of the 2010s variety were the highest among the tested varieties. The main reason was that the 2010s variety prolonged T1 by 6.91–32.50% and improved the V2 and V3 by 6.49–13.89%, 4.55–15.00% compared with 1970–2000s variety.

Subsoiling tillage increased the Gmean by improving the filling rate and prolonging the grain filling duration in the rapidly increasing period and slowly increasing period, and so the grain weight increased significantly. In the rapidly increasing period, the filling rate of all tested varieties improved by 0.00–2.44%, the grain filling duration extended by 1.20–4.00%, and those two parameters increased by 0.00–2.44% and 1.20–4.00% respectively in the slowly increasing period. Moreover, the subsoiling helped the Rmax increase by1.23–4.86%, and Tmax prolong by 4.04–5.80%. Meanwhile the filling rate of the rapidly increasing period of the 2010s variety was more sensitive to subsoiling tillage in comparison with other trial varieties.
